# Monte Carlo Simulation on Adiabatic Ensembles and a Genetic Algorithm

**DOI:** 10.3390/e27060565

**Published:** 2025-05-27

**Authors:** Fernando M. S. Silva Fernandes

**Affiliations:** Centro de Química Estrutural, Institute of Molecular Sciences, Departamento de Química e Bioquímica, Faculdade de Ciências, Universidade de Lisboa, 1749-016 Lisboa, Portugal; fmfernandes@ciencias.ulisboa.pt

**Keywords:** Monte Carlo simulation, adiabatic ensemble, entropy, genetic algorithm

## Abstract

This paper concerns interactive Monte Carlo simulations for adiabatic ensembles and a genetic algorithm to research and educational contexts. In the Introduction, we discuss some concepts of thermodynamics, statistical mechanics and ensembles relevant to molecular simulations. The second and third sections of the paper comprise two programs in JavaScript regarding (*i*) argon in the grand-isobaric ensemble focusing on the direct calculation of entropy, vapor–liquid equilibria and radial distribution functions and (*ii*) an ideal system of quantized harmonic oscillators in the microcanonical ensemble for the determination of the entropy and Boltzmann distribution, also including the definition of Boltzmann and Gibbs entropies relative to classical systems. The fourth section is concerned with a genetic algorithm program in Java, as a pedagogical alternative to introduce the Second Law of Thermodynamics, which summarizes artificial intelligence methods and the cumulative selection process in biogenesis.

## 1. Introduction—Ensembles and Averages

In the 1950s, Metropolis et al. [1] and Alder et al. [2] performed the first Monte Carlo and molecular dynamics simulations with particles interacting through hard and square-well potentials. Since then, the steady increase of computational resources provided the means for extending simulation methods to equilibrium and non-equilibrium thermodynamics and statistical mechanics, quantum mechanics, chemical kinetics [2,3,4,5,6,7,8,9] and artificial intelligence and information theory [10,11,12].

Molecular dynamics determine the time evolution of microstates through the integration of molecular motion equations and the time averages of the mechanical properties over successive microstates. The classical microstates are defined by the positions and momenta of the molecules and, as the motion equations are time reversible, statistical mechanics assume that after a long enough time, the system will eventually flow close to nearly all microstates. For quantum dynamical systems, the microstates are defined in terms of wave functions or the isomorphism between quantum and classical particles, and their evolution is followed through ab initio or path integral methods (Allen et al. [3], Ramalho et al. [13]).

The time average of a classic mechanical property *X* is

(1) $$<X{>}_{time}=\frac{1}{M}\sum_{\nu =1}^{M}X_{\nu }$$

where *M* is the number of microstates along the simulation and $X_{\nu }$ is the property of microstate ν. Mechanical properties (like potential energy and pressure) are defined for every microstate by the positions and momenta of the molecules, but statistical properties (like entropy and free energies) depend on all microstates consistent with the constraints of the system (the so-called multiplicity of the macrostate).

Equation (1) can be rewritten as follows:

(2) $$<X{>}_{time}=\sum_{\nu }\frac{n_{\nu }}{M}X_{\nu }=\sum_{\nu }P_{\nu }X_{\nu }$$

where $n_{\nu }$ is the number of times that the system flows close to microstate ν and $P_{\nu }=n_{\nu }/M$ is the microstate probability. The probabilities in the last equation suggest that each microstate ν may be generated by moving the particles randomly and weighting the property $X_{\nu }$ with probability $P_{\nu }$ to estimate ensemble averages. In turn, statistical mechanics assume the equivalence of ensemble and time averages:

(3) $$<X{>}_{ensemble}=\sum_{\nu }P_{\nu }X_{\nu }=<X{>}_{time}$$

This is the basis of the Monte Carlo method, which, in assuming the equality of ensemble and time averages, is equivalent to the molecular dynamics method. However, there is a crucial difference. Molecular dynamics, a deterministic method, integrate motion equations, and the microstates are defined by the positions and momenta of the particles as a function of time. Monte Carlo, a stochastic method, generates an ensemble of random microstates not requiring the integration of motion equations. As such, the microstates do not depend upon time and, in general, are only defined by the random molecular positions.

The ensemble concept was introduced by Gibbs as considering a large number of virtual copies of a system all at once with each copy representing a possible microstate that the real system might be in. Based on this concept, Gibbs founded the statistical mechanics of ensembles (Klein [14]).

As for the equivalence of ensemble and time averages, Chandler [15] writes:

The equivalence of a time average and an ensemble average, while sounding reasonable, is not at all trivial. Dynamical systems that obey this equivalence are said to be *ergodic*. It is difficult, in general, to establish the principle of ergodicity, though we believe it holds for all many-body systems encountered in nature. (It is often true for very small systems too, such as polyatomic molecules. Indeed, the basis of the standard theories of unimolecular kinetics rests on the assumed ergodic nature of intramolecular dynamics).

Recently, Baldovin et al. [16] pointed out that only a limited number of systems can be proved to be ergodic, and that weak forms of ergodicity hold even in systems that are not ergodic at the microscopic scale provided that extensive observables are considered. Nevertheless, most simulations of many-body systems by molecular dynamics and Monte Carlo have excellent agreement with experimental data, which is just an indication of their ergodicity. Even so, ergodic problems may arise when a system eventually remains stuck in phase space regions (Allen et al. [3], Frenkel et al. [5]).

It is also worth noting that the uncertainties of perturbations or randomness, ever present in many-body systems, are taken into account by semiclassical statistical mechanics, dividing phase space into cells with dimensions of Planck’s constant, *h*. According to Kelly [17], if *h* → 0, then the entropy, *S* → *∞*.

The statistical ensembles and the microstate probabilities are defined by the thermodynamic constraints. For example, the grand-isobaric adiabatic ensemble is constrained by the chemical potential (μ), pressure (*p*) and Ray energy (*R*), which remain constant, while the volume *V* and the number of particles *N* fluctuate. After integrating the kinetic contribution to the Hamiltonian, the probability of a microstate containing *N* particles with coordinates q in a volume *V* is (Desgranges et al. [18]):

(4) $$P_{\nu }\left(q,N,V\right)=\frac{{\left(bV\right)}^{N}}{\Gamma (3N/2)Q(\mu ,p,R)}{\left[R-pV+\mu N-U\left(q\right)\right]}^{3N/2-1}$$

being $b={\left(2\pi m/h^{2}\right)}^{3/2}$, *m* the molecular mass, *h* the Planck constant, Γ() the gamma function, $Q\left(\mu ,p,R\right)$ the ensemble partition function and $U\left(q\right)$ the potential energy.

The microstates are generated by four moves: random displacements of particles; the insertion of a new particle at a random position in the system; the removal of a particle chosen at random; and a random change of the system volume. The simulations can be implemented in two different ways:

(1) Accept all microstates, weight them with probability $P_{\nu }$ and calculate the averages according to Equation (3). This way is, in general, of poor statistical efficiency since most generated microstates have small contributions to the averages. Moreover, it would require the calculation of the partition function, which is not suitable for this particular model.
(2) Accept a new microstate (*n*) from an old one (*o*) with probability $acc=min\left[1,\frac{P\left(n\right)}{P\left(0\right)}\right]$ and calculate the averages by an arithmetic mean. This is the seminal idea of Metropolis et al. [1] that provides an importance sampling because the accepted microstates are the ones that most contribute to the averages. On the other hand, the quotient of acc eliminates the partition function. This method, dubbed Metropolis Monte Carlo, is implemented in the model of Section 2.

In addition to the grand-isobaric ensemble, other adiabatic and non-adiabatic ensembles are widely used in molecular simulations either by Monte Carlo or molecular dynamics, to wit: microcanonical (E,V,N), isoenthalpic-isobaric (H,p,N), grand-isochoric (μ,V,L), canonical (T,V,N), grand-canonical (μ,V,T) and isobaric-isothermal (p,T,N). The formalism of equilibrium statistical ensembles can be derived through Legendre and Laplace transforms (Pearson et al. [19], Graben et al. [20,21]). As these transforms preserve all thermodynamic information of the systems, the equilibrium statistical ensembles are equivalent in the thermodynamic limit. However, the choice of the convenient ensemble depends on the thermodynamic constraints of the system to be simulated, and the methods eventually required to specific ends, e.g., nonphysical sampling, density of states estimation, phase equilibria, time-dependent properties and open systems. For example, the simulations of open systems in the grand-isobaric, grand-isochoric and grand-canonical ensembles require the insertion and deletion of particles, which is a problem mainly at high densities because the majority of the attempted moves are rejected. Then, additional methods to circumvent the problem and accelerate the computations are generally applied (Allen et al. [3], Site et al. [22]). Grand-canonical simulations have been carried out by Monte Carlo and molecular dynamics (Sadus [4]), but most grand-adiabatic simulations, until now, were performed by Monte Carlo. One of the first, if not the first, molecular dynamics simulation in the grand-isochoric (μ,V,L) was completed by Çagin et al. [23]. Their model, also applied to the grand-canonical molecular dynamics, is conceived such that the time averages over the molecular trajectories are equivalent to ensemble averages. The Legendre and Laplace transforms show that each statistical ensemble is characterized by a thermodynamic potential function (also called fundamental or characteristic function) and a partition function, which is connected by a generalized Boltzmann equation. For instance, the potential function of the four adiabatic ensembles is the entropy, and for the grand-canonical ensemble, it is the grand-energy J=A−μN, where *A* is the Helmholtz energy. Once the respective partition functions are determined, analytically or numerically, the properties of the systems can be calculated through thermodynamic derivatives over the potential and partition functions, as in the works of Desgranges et al. [24], Ströker et al. [25] and Lustig [26] in the contexts of the grand-canonical and grand-adiabatic ensembles (see Section 2) and the microcanonical ensemble (see Section 3).

The number of molecules in bulk systems is of the order of Avogadro’s number, $10^{23}$ (the so-called thermodynamic limit). Then, if the aim is to calculate bulk properties, boundary conditions and long-range corrections are generally applied to mitigate inevitable effects (e.g., surface effects), since the number of particles in simulations is rather small relative to the thermodynamic limit. However, small systems far from the thermodynamic limit, beyond the interest in statistical mechanics, are a relevant subject due to the increase in resolution in the observation and manipulation of biological and man-made objects at micro and nano-scales (Puglisi et al. [27], Rodrigues et al. [28]).

## 2. Grand-Isobaric Ensemble Model

### 2.1. Introduction

The grand-isobaric adiabatic ensemble (μ,p,R) describes a system enclosed by adiabatic, movable and porous walls which hold as constants the chemical potential (μ), the pressure (*p*) and the Ray energy (R = H −μ * N) where *H* is the enthalpy and *N* is the number of molecules.

The entropy is a homogeneous function of first order expressed by Euler’s relation (Callen [29]), that is,

(5) $$S=\left(E+pV-\mu N\right)/T=\left(H-\mu N\right)/T=R/T$$

being E=K+U the internal energy, *K* the kinetic energy, *U* the potential energy and *T* the temperature. While μ, *p* and *R* remain constant, *T*, *N* and other properties will fluctuate whose averages are calculated along the simulations. Therefore, the ensemble average of the entropy in the grand-isobaric ensemble can be calculated directly as follows:

(6) $$<S\left(\mu ,p,R\right)>=R/<T>$$

The first Monte Carlo simulations in the grand-isobaric ensemble were performed by Ray and Wolf [30] for liquid palladium as a demonstration that the ensemble enables a direct route to the entropy according to Equation (6), as well as, other particular features.

Recently, Desgranges and Delhommelle [18] applied grand-isobaric Monte Carlo simulations to argon and copper, and they extended the method to binary mixtures of argon–neon and copper–silver [31]. They also studied argon and carbon dioxide combining the Wang–Landau sampling with expanded grand-canonical simulations, focusing on the vapor–liquid equilibria and surface tension [24]. After this work, the authors applied the Wang–Landau method in a considerable sequence of papers on a wide range of properties, even combining the Wang–Landau sampling with machine learning [32]. This set of papers is easily found in the internet—some for free download.

Furthermore, Ströker and Meier [25] made an analytical study of the grand-isochoric (μ,V,L) and grand-isobaric (μ,p,R) ensembles, deriving expressions for different thermodynamic properties, which are validated by Monte Carlo simulations of the Lennard–Jones fluid.

### 2.2. Program and Algorithm

The model simulates by the Metropolis Monte Carlo method a set of particles interacting through the Lennard–Jones potential parametrized for argon, with cubic boundaries, minimum-image convention and long-range corrections.

The JavaScript program is available at Grand-Isobaric Ensemble (https://www.compadre.org/osp/items/detail.cfm?ID=16805, accessed on 1 May 2025) where it can be directly executed as a ready-to-run example, or as a ready-to-edit example where the user can inspect the code and the graphical objects implementation. The description of the model with suggested activities is also available in the PDF document.

**Figure 1:**
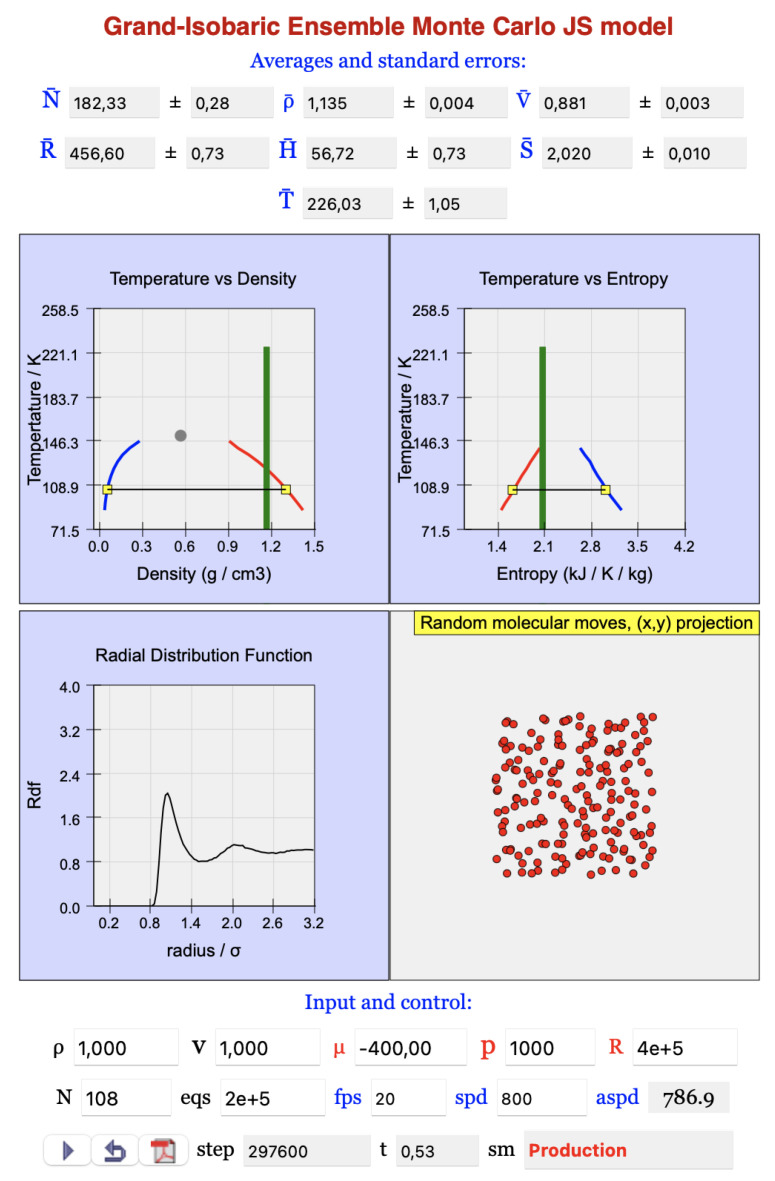
Interactive display of the grand-isobaric ensemble model.

Figure 1 shows the interactive display of the JavaScript model when the ready-to-run option is chosen; for the ready-to-edit option, the display has an additional window with the code and the graphical objects implementation.

From an initial configuration, the Monte Carlo moves are attempted according to the following rates:

- 1% random changes of the volume;
- 33% displacements of particles selected at random;
- 33% insertions of new particles into locations selected at random;
- 33% removals of particles selected at random.

The acceptance probability formulae for each of the attempted steps are explicitly given in the paper of Desgranges et al. [18] according to the Metropolis recipe.

The entropy is directly calculated through Equation (6):

$$<S\left(\mu ,p,R\right)>=R/<T>$$

The average temperature <T> is given by the equipartition theorem:

(7) $$<T>=2<K>/\left(3<N>k_{B}\right)$$

where $k_{B}$ is Boltzmann’s constant, and

(8) <K>=R−<U>−p<V>+μ<N>

The temperature is not a constant property of the ensemble, but it can be controlled through the chemical potential. In fact, for a given Ray energy and pressure, if the chemical potential increases, the temperature, specific volume and entropy will decrease. Then, by decreasing the temperature (i.e., increasing the chemical potential) stepwise, at a fixed pressure, the vapor–liquid coexistence curves can be mapped [18] as an alternative to other Monte Carlo and molecular dynamics methods (Desgranges et al. [24], Wilding [33], Errington [34], Fernandes et al. [35], Heyes [36]).

The (μ,p,R) simulations reveal a particular feature: “for a given pressure and chemical potential, if the Ray energy is increased or decreased, the volume and the number of molecules scale accordingly so that the averages of the intensive properties (density, specific volume, temperature) and the properties per molecule (R/N, H/N, S/N) remain approximately constant within the standard errors”. This allows a choice of the convenient values of the Ray energy and the number of particles for the simulations, which is essential in the definition of the initial conditions and to improve the statistics.

The program was tested against the simulation results of Desgranges et al. and experimental data for argon [18,24] following the activities described in the PDF document.

## 3. Microcanonical Ensemble Model

### 3.1. Introduction

The microcanonical ensemble (E,V,N) is another adiabatic ensemble that describes a system enclosed by adiabatic, immovable and non-porous walls (i.e., an isolated system) which hold as constants the internal energy *E*, the volume *V*, and the number of molecules *N*.

This model, inspired by the pedagogical article of Schettler [37], simulates an isolated system of 1000 distinguishable and non-interacting molecules modeled like quantized harmonic oscillators. Through random molecular jumps between the energy levels of the oscillators, the entropy of the system increases toward a maximum, which is consistent with the constraints of the system, and Boltzmann’s distribution is approached. That is, the system behaves according to the Second Law of Thermodynamics: “for an isolated system undergoing changes the entropy evolves to a maximum value at the equilibrium state, i.e., ΔS≥0”.

The entropy is given by the Boltzmann equation:

(9) $$S/k_{B}=ln\;W$$

being $k_{B}$ Boltzmann’s constant, ln the natural logarithm function and *W* the number of microstates of a macrostate, i.e., the multiplicity of the macrostate.

The distribution of the molecules over the energy levels is followed through the Boltzmann distribution:

(10) $$n_{i}/N=exp\left(-\beta \;\varepsilon_{i}\right)/\sum_{i=0}^{N}exp\left(-\beta \;\varepsilon_{i}\right)$$

where $n_{i}$ is the number of molecules at level *i* of energy $\varepsilon_{i}$, $\beta =1/k_{B}T$, *T* is the temperature and *N* is the total number of molecules.

For distinguishable, non-interacting molecules with non-degenerate energy levels, the multiplicity of the macrostates is

(11) $$W=\frac{N!}{n_{0}!n_{1}!\;\ldots n_{i}\;!}$$

as a function of the factorials of the total number of particles and the number of particles at each energy level.

The quantized energies of the levels, $\epsilon_{i}$, are given by $\varepsilon_{i}=\left(i+1/2\right)h\nu$; $i=0,1,\ldots n_{i}$ where *h* is Planck’s constant and ν is the oscillator frequency.

The energy levels are equally spaced $\left(\varepsilon_{i+1}-\varepsilon_{i}=h\upsilon \right)$, and taking the zero-point energy (1/2 hν) as a reference, there is no ambiguity in denoting the level energies by 0,1,2,… That is, energy “0” corresponds to 1/2 hν, energy “1” to hν, energy “2” to 2 hν, and so forth. Defining the unit of energy (u.e.) = hν, then the energy of a molecule at any level, for example level 10, is 10 u.e. Moreover, $k_{B}$ is taken as the unit of entropy, i.e, $k_{B}=1$, so Equation (9) becomes, S=lnW.

### 3.2. Program and Algorithm

The JavaScript program is available at Entropy and Boltzmann’s Distribution (https://www.compadre.org/osp/items/detail.cfm?ID=16724, accessed on 1 May 2025) where it can be directly executed as a ready-to-run example or as a ready-to-edit example where the user can inspect the code and the graphical objects implementation.

**Figure 2:**
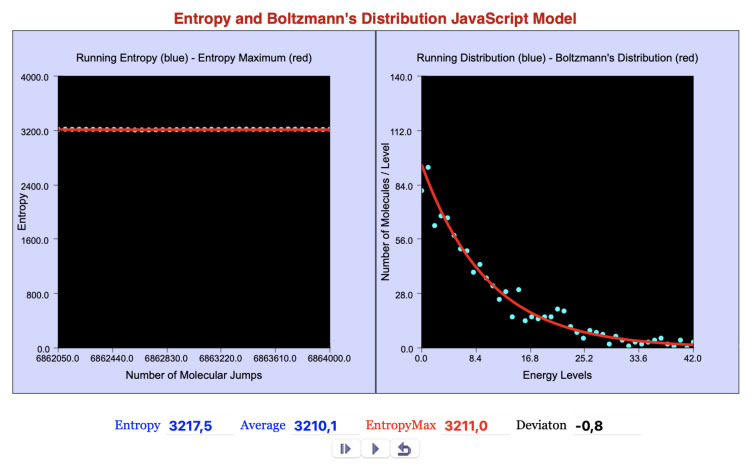
Interactive display of the microcanonical ensemble model.

Figure 2 shows the interactive display of the JavaScript model when the ready-to-run option is chosen; for the ready-to-edit option, the display has an additional window with the code and the graphical objects implementation.

(a) Initially, 1000 molecules are set at level 10. Therefore, the total energy of the system is 1000 * 10 u.e.
(b) Two different molecules are successively chosen at random. Then, one of the molecules jumps to the next higher level, increasing the energy by 1 u.e.; the other molecule jumps to the next lower level, decreasing the energy by the same amount. Thus, the total energy of the system is kept constant.
(c) The running entropy is calculated by successive increments of 2000 jumps, and its average displayed every 50 jumps.
(d) Calculation of β (Dessaux et al. [38]), for Boltzmann distribution and entropy maximum at equilibrium (red lines in the displays) evaluated at initialization.

Let us define x=exp(−β), where 0<x<1 and the partition function $Z=\sum_{i}x^{\varepsilon_{i}}$.

The average energy per molecule is $\sum_{i}n_{i}\epsilon_{\prime }/N$ = $\sum_{i}\epsilon_{i}x^{\epsilon_{i}}/Z$ = 10 u.e, since initially, the 1000 molecules were set at level 10, and the total energy is kept constant.

Preliminary tests have shown that the energy levels worth considering for this model are the first 41. Thus, expanding the last equation to the first 41 levels:

$$x\left(9+8x+7x^{2}+\ldots x^{8}-x^{10}-2x^{11}-\ldots 31x^{40}\right)=0$$

whose numerical root (0<x<1) is *x*= 0.907, whence β = 0.0976.

### 3.3. Comments

(1) As Schettler [37] also commented, the model with its unitary energy changes (see (b) of the algorithm) does not simulate real processes for which multiple units of energy changes are allowed. However, the agreement of the simulation with the Second Law of Thermodynamics raises the question of how the molecular distribution is independent of the details of the jump process but depends only on the equilibrium state being attainable and upon microscopic reversibility, i.e., that the reverse process must be as probable as the forward one.

Entropy and the Second Law of Thermodynamics is the matter of various pedagogical articles, e.g., Moore et al. [39], Timberlake [40], and Salagaram et al. [41] with computational models focusing the entropy. Baierlein [42] focused on a pedagogical alternative to introduce the Second Law and entropy, contributing to the discussion of how to teach thermodynamics. Ben-Naim [43] interprets entropy changes of mixing and demixing processes in terms of Shannon’s measure of information. Lieb et al. [44] take a non-statistical mechanics view of entropy and the Second Law, which is the basis of an axiomatic foundation for thermodynamics. Wolfram [45] resorts to a non-molecular model (the rule 30 cellular automaton), conveying a deep thought:

“It is all a story of the interplay between underlying computational irreducibility and our nature as computationally bounded observers. Other observers—or even our own future technology—might see things differently. But at least for us now, the ubiquity of computational irreducibility leads inexorably to the generation of behavior that we—with our computationally bounded nature—will read as “random”. We might start from something highly ordered (like gas molecules all in the corner of a box) but soon—at least as far as we are concerned—it will typically seem to “randomize” just as the Second Law implies”.

(2) The fluctuations of entropy and number of particles at energy levels, observed in the displays, do not mean changes of entropy with time, which is not a variable in this Monte Carlo model. However, Monte Carlo fluctuations mimic the fluctuations in systems where time is involved. For example, time is an explicit variable in molecular dynamics, and the simulations can calculate, e.g., speed distribution functions. Monte Carlo can accomplish the same by randomly sampling the molecular positions and velocities (Fernandes et al. [46]). The results are equivalent, in accordance to the statistical mechanics assumption of the equality of time and ensemble averages (see Section 1).

(3) There is a difference between the Monte Carlo sampling in this microcanonical model (MCM) and the grand-isobaric model (GIM). In MCM, each non-interacting particle is identified by an integer number and the energy level where it is located, translational degrees of freedom are not involved, and the volume can be considered arbitrarily constant. In GIM, the particles interact, have translational degrees of freedom, and are identified by their coordinates, and the volume and number of particles change along the simulations. In MCM, the molecular jumps are always accepted when two different particles are randomly chosen, and the algorithm ensures the conservation of internal energy and number of particles. In GIM, the four moves are also generated randomly, but their acceptance is decided by the Metropolis importance sampling. A question turns out: why not implement the Metropolis sampling in MCM, or is it already implicit? Please look at the code of function jumps () in the ready-to-edit example at the program site and calculate the probability of selecting two different non-interacting particles (think about two dice with 1000 faces).

### 3.4. Density of States—Boltzmann and Gibbs Entropies

Counting the number of microstates by factorials, like Equation (11), is impracticable for many-body thermodynamic systems whose spacing between levels is so small as to form a continuum. For them, the entropy and other properties are calculated through the semiclassical phase space volume and phase space density.

The phase space volume Ω encloses the microstates with energy less than or equal to *E* and the phase space density ω = (number of microstates within a very narrow phase space shell of thickness *E - dE)/dE*, which is defined by the following equations:

(12) $$\omega \left(E,V,N\right)={\left(N!\;h^{3N}\right)}^{-1}\;\int \int \;\delta \left[E-H\left(q^{N},p^{N}\right)\right]dq^{N}dp^{N}$$

(13) $$\Omega \left(E,V,N\right)={\left(N!\;h^{3N}\right)}^{-1}\;\int \int \;\Theta \left[E-H\left(q^{N},p^{N}\right)\right]dq^{N}dp^{N}$$

where $h,\delta ,\Theta ,H,q^{N},and\;p^{N}$ are, respectively, the Planck constant, the Dirac delta function, the unit step function, the Hamiltonian and the coordinates and momenta of the *N* particles. N! corrects the distinguishability of the particles implicit in the integrals, and $h^{3N}$ are the uncertainties of order *h* ($dqdp=h^{3}$) referred to in Section 1. The delta and unit step functions trace the difference between the two definitions which are just expressions of the microcanonical partition function.

These equations define Boltzmann entropy (also called surface entropy), $S_{B}$, and Gibbs entropy (also called volume entropy), $S_{G}$:

(14) $$S_{B}/k_{B}=ln\;\omega \left(E,V,N\right)$$

(15) $$S_{G}/k_{B}=ln\;\Omega \left(E,V,N\right)$$

The phase space has a startling property: “in the thermodynamic limit, the phase space volume and the phase space density are equivalent”. Callen [29] expressed this as: “In an imaginary world of high dimensionality there would be an automatic and perpetual potato famine, for the skin of a potato would occupy essentially its entire volume!”

However, since the number of particles in molecular simulations is rather small relative to the thermodynamic limit, the difference between Boltzmann entropy and Gibbs entropy has been analyzed. Lustig [26] conducted a detailed study by mathematical analysis and simulations of small systems (with and without boundary conditions). He concluded that the Boltzmann definition is more rigorous than the Gibbs definition, and that errors of order lnω/N and lnΩ/N extrapolated to the thermodynamic limit (N→∞) turn the two definitions numerically equivalent. The thermodynamic potential function of the microcanonical ensemble is the entropy, and the partition function is given by Equations (12) or (13). The relation between the two functions is expressed by Equations (14) or (15). Once the partition function is determined, the thermodynamic properties are calculated through thermodynamic derivatives. A similar study and method was implemented by Ströker et al. [25] for the grand-isochoric and grand-isobaric adiabatic ensembles.

Beyond that, Boltzmann and Gibbs entropies have been the subject of extensive debates focusing on the strength and weakness of their definitions and the question of absolute negative temperatures (Frenkel et al. [47], Swendsen [48], Shirts [49], Rajan [50]).

Let us see two ways for simulations:

(i) Following Lustig’s method, derive expressions for the thermodynamic properties and apply them.
(ii) From Equation (13), the probability of a microstate is
  (16) $$P_{\Theta }\left(E,V,N\right)=\frac{\Theta \left[E-H\left(q^{N},p^{N}\right)\right]}{\left(N!\;h^{3N}\right)\;\int \int \;\Theta \left[E-H\left(q^{N},p^{N}\right)\right]dq^{N}dp^{N}}$$

Apply a Metropolis Monte Carlo recipe, either sampling the particle coordinates, after integrating out the kinetic part of the hamiltonian (Ray [51]), or sampling the coordinates and momenta (Fernandes et al. [46]).

The probability of a microstate from Equation (12) is similar to that of Equation (16) inserting the delta function instead of the unit step function. All microstates enclosed by phase space density and phase space volume have energy *E* in the thermodynamic limit. Therefore, from the definitions of the delta and unit step functions, the probability of any microstate is 1/ω=1/Ω; that is, the microstates have equal probability, as postulated by the microcanonical statistical mechanics (isolated systems). However, as the number of molecules in the simulations is rather small relative to the thermodynamic limit, then: 1/Ω≈1/ω i.e., $P_{\Theta }\;\approx \;P_{\delta }$.

## 4. A Genetic Algorithm Model

### 4.1. Introduction

Shakespeare and monkeys have inspired scientists and philosophers since a long time ago. Let us see how they enter into the play:

The Infinite Monkey Theorem [52], traced to the mathematician Émile Borel, or even before, states that “a monkey hitting at random on a typewriter keyboard for an infinite amount of time will almost surely type the complete works of William Shakespeare”.

Recently, the mathematicians Stephen Woodcock and Jay Falleta considered the Finite Monkeys Theorem [53] and demonstrated that the Infinite Monkey Theorem is misleading in our finite universe.

In the same vein, by the way of the conversion of thermal energy to work, the physical chemist Henry Bent wrote [54]:

“Complete conversion of thermal energy to work is virtually impossible; it is about as likely as the transcription of Shakespeare’s complete works by a tribe of wild monkeys punching randomly on a set of typewriters. In all likelihood no one will ever witness either event; moreover, as once noted, were someone to see thermal energy completely converted to work, or monkey-business turn out Shakespeare, he would probably not believe it”.

Then, considering the process that has as its net effect removal, from a 400 K thermal reservoir, of 1200 calories and the raising of a weight by an equivalent amount, he calculated the respective probability:

$10^{-400,000,000,000,000,000,000,000}$ to 1, that is, one chance in $10^{400,000,000,000,000,000,000,000}$, and the negative change of entropy (of the universe in this process), ΔS=−3 cal/deg. These results verify the Kelvin–Planck formulation of the Second Law of Thermodynamics, and they do not feed the ’dream’ of a perpetual machine of the second kind.

In turn, the biologist Richard Dawkins [55] explained how complex molecules essential to life, like hemoglobin, may be synthesized by cumulative selection, which is a process that despite having random mutations is not at sheer chance. To this end, he introduced the evolutionary program Weasel, in Basic and Pascal, to reproduce Hamlet sentence: ME THINKS IT IS LIKE A WEASEL. Since then, the Weasel program has been a source of different versions, e.g., James Freeman’s genetic algorithm in *Mathematica* [56], and a Python version posted by GitHub [57].

The present genetic algorithm in Java is just an extension of Dawkins’s and Freeman’s programs. It generates successive strings of alphanumeric characters from a random population, mutating the strings (without crossover) with a fixed probability in order to match the target string previously chosen by the program user. Strings are kept or discarded accordingly to their fitness to the target string.

Genetic algorithms are optimization methods based on evolutionary principles widely used in exact and life sciences, artificial intelligence (AI) and industry (Cartwright [11,58]). In 1995, Cartwright wrote: “AI is a young and largely unexplored subject. Despite its short history, however, its potential is unmistakable. AI will be a central part of the chemist’s arsenal within two decades”.

For example, among AI methods are the artificial neural networks, which are particularly relevant for physics, chemistry, production processes and machine learning (Zupan et al. [59], Desgranges et al. [32,60], Latino et al. [61]), and the genetic programing, based on genetic algorithms, that Cartwright called the Holy Grail of scientific computing [11]. Recently, Schwalbe-Koda et al. [62] reported the relevance of information theory in machine learning-driven simulations unifying materials modeling and statistical mechanics.

### 4.2. Program, Algorithm, Guide and Suggested Activities

The Java program ’GeneticAlgorithmModel.jar’ is available at Supplementary Materials. After downloading the jar file, it runs under Unix, Linux or Windows, either double clicking on the file, or from the console by the command: jar -jar GeneticAlgorithmModel.jar, providing that the Java Runtime Environment (JRE) is installed (at least version 1.8 is recommended).

**Figure 3:**
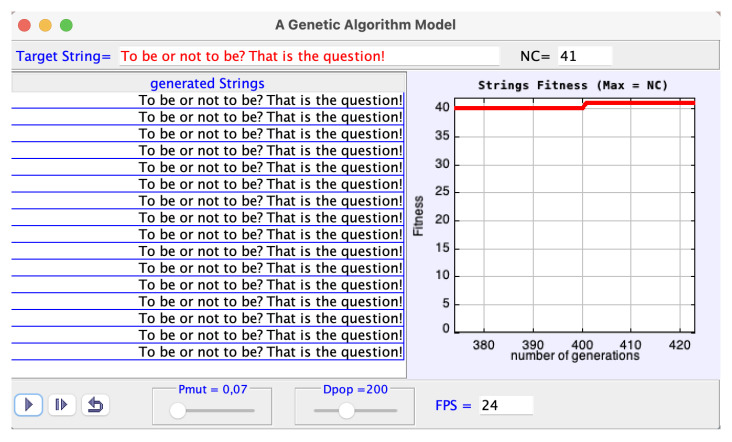
Interactive display of the genetic algorithm model.

Figure 3 shows the interactive display of the Java model which is complemented by another window with additional information.

Let us take the default string just as an example of the algorithm steps executed by the program:

The default target string, already inscribed, is: To be or not to be? That is the question! It has 41 characters (NC = 41) counting the blank spaces.

(1) Set up a random population of strings, with dimension Dpop = 200, each string with 41 random characters.
(2) Determine the fitness of each random string; that is, calculate of the number of characters that eventually coincide with the ones of the target string in their values and respective positions.
(3) Select the string with greatest fitness (“the parent”) and display.
(4) Mutate parent’s characters with probability Pmut = 0.07 to generate a new population of dimension 200.
(5) Go to (3) until the target string is eventually reproduced.

**Guide and Examples**

- When the simulation is paused, the user can change the target string, Pmut in the interval [0.0, 1.0] and Dpop in the interval [1, 500] by the respective sliders. As for the FPS field (the number of frames per second), it controls the speed of the displays. It accepts values [1, 24], and 100, and it is editable when the run is paused.
- After editing a data field (target string or/and FPS), the enter key must be pressed; otherwise, the field is not actualized. The same applies after moving the sliders.
- The mouse over a label or button displays a tooltip.
- The program processes only printable characters with ASCII DEC codes from 32 to 126 (https://www.ascii-code.com, accessed on 1 May 2025).
- For Pmut = 0.0, there are no mutations, so the strings do not change.
- For Pmut = 1.0, the characters of the parent are mutated uniformly at random, i.e., at sheer chance, so the successive strings do not converge to the target.
- The default value Pmut = 0.07 leads to a good convergence to the default target.
- If the target is longer than the default one, for example: Shakespeare: To be or not to be? That is the question! is better reproduced with Dpop = 400 even with Pmut = 0.07.
- The shorter target: tobeornottobe is well reproduced with Pmut = 0.1 and Dpop = 100.

**Note**: The program handles relatively short strings and Dpop, and it does not include crossover. If the target and Dpop are too long, crossover and a more extended display are recommended (see Activity 4).

**Suggested Activities**

(1) The total number of words in Shakespeare works (https://www.opensourceshakespeare.org/statistics/, accessed on 1 May 2025) is 884,421. The number of English characters per word is about 6 (Wikipedia).
  Henry Bent another comparison [54]:
  “At room temperature, for example, the conversion of a single calorie of thermal energy completely into potential energy is a less likely event than the production of Shakespeare’s complete works fifteen quadrillion times in succession without error by a tribe of wild monkeys punching randomly on a set of typewriters”.
  (a) Calculate the probability of Bent’s monkey metaphor (the Infinite and Finite Monkeys Theorems, cited in Section 4.1, can help).
  (b) Compare with the probability he calculated for the complete conversion of thermal energy to work (see Section 4.1).
(2) Reproduce Hamlet’s sentence: ME THINKS IT IS LIKE A WEASEL with different values of Pmut, Dpop and FPS, looking at the fitness and number of generations.
(3) Reproduce the target: CH3-CH2-CH2-CH2-CH2-CH2-CH2-CH2-CH2-CH2-NH2 with different values of Pmut, Dpop and FPS, looking at the fitness and number of generations.
(4) Install Java 6.02 modeling tool from Easy Java Simulations (https://www.um.es/fem/Ejs/, accessed on 1 May 2025) in a computer under Unix, Linux or Windows. Run the GeneticAlgortihmModel.jar and follow the instructions in the respective Intro Page to load the program source, automatically named GeneticAlgorithmModel.ejs, into the modeling tool. Then, the code can be inspected and eventually modified. To include crossover, see, e.g, Cartwright [16,58]. In any case, search for the Guides at Easy Java Simulations (https://www.compadre.org/osp/?, accessed on 1 May 2025). This is essential reading, especially by users who have never used it. The modeling tool and respective paths should be configured after its installation.

### 4.3. Comments

(1) The algorithm steps in Section 4.2. show that the successive populations are generated by mutations, with probability Pmut, from a “parent” i.e., the string with the greatest fitness to the target. If Pmut < 1, the target should be reproduced sooner or later depending on the values of Pmut and Dpop. This is just a simple example of the cumulative selection process which, despite having random mutations, is not at sheer chance because Pmut < 1.
(2) If Pmut = 1, the process is at sheer chance, and the strings chosen by the user are not reproduced, which is in accordance with the improbability of the monkey business and the complete conversion of thermal energy into work.
(3) This Monte Carlo simulation, unlike the grand-isobaric and microcanonical models, does not deal with molecules and ensemble averages. As such, it is not a molecular simulation but rather just an example of an optimization method based on evolutionary principles; however, it is related to physical and biological properties.
(4) The purpose of the simulations is defined at start by the program user (target, Pmut and Dpop). But what about the purpose of natural abiogenesis and biogenesis? Is there no purpose whatsoever, or there is a previous design? These questions are the root of extensive debates (Dawkins [55], Dembski et al. [63], Scientific American [64]); however, it is not in the ambit of this article.
(5) Nevertheless, there is a point about the Second Law of Thermodynamics. Some creationists claim that the 2nd Law contradicts the evolution of species, because the order of the living structures relative to the supposed disorder of their ancestors decreases the entropy of the universe. The creationists’ argument is untrue (Styer [65]). Indeed, it does not account for the role of the environment. In the context of natural evolution, genetic algorithms have also been criticized (Dembski et al. [63]).
(6) Kondepudi et al. [66] revealed that some dissipative structures exhibit organism-like behavior in electrically and chemically driven systems. The highly complex behavior of these systems shows the time evolution to states of higher entropy production. Taking these systems as an example, they present some concepts that give an understanding of biological organisms and their evolution.
(7) Čápek et al. [67], regarding the challenges of the Second Law of Thermodynamics, cite twenty-one formulations of the Law, some not invoking the concept of entropy.

## 5. Conclusions

Our results from the grand-isobaric ensemble agree well with other simulations and experimental data for argon. The direct computation of the entropy, one of the objectives of the model, requires only the Ray energy input by the program user and the average temperature calculated along the simulations. Extra routines for the calculation of pressures and chemical potentials are unnecessary because their values given at initialization remain constant along the simulations. The mapping of vapor–liquid coexistence curves is relatively easy through the control of the input pressures and chemical potentials, as an alternative, for example, to Gibbs ensemble with which our results are in good accordance. Gibbs ensemble ensures, by definition, the equality of the chemical potentials and pressures along the coexistence lines. Even so, it is always convenient to confirm it by extra routines. Moreover, the existence of two simulation boxes may be problematic mainly when the critical point is approached and one of the boxes may eventually become empty. The displays of the radial distribution functions and animations of the random molecular moves visualize the changes of the particles’ distribution and the box volume due to the automatic increase or decrease in the number of particles along the simulations until the systems attain the equilibrium state.

The microcanonical ensemble program simulates an unrealistic system not to be tested against experimental data. However, its accordance with the Second Law of Thermodynamics suggests that the molecular distribution is independent of the details of the jump process but depends only on the equilibrium state being attainable and upon microscopic reversibility. On the other hand, it brings about the problem of determining the multiplicity of macrostates for many-body systems when factorial expressions are impracticable, the consequent definition of Boltzmann and Gibbs entropies, the general method to calculate the properties of the systems through the connection between the thermodynamic potentials and the partition functions, and the interpretation of Monte Carlo versus molecular dynamics fluctuations.

As for the genetic algorithm model, despite not including the majority of the genetic algorithms resources, it is suitable to test other simple genetic algorithms, and it is also suitable as a pedagogical alternative to introduce the improbability of the complete conversion of thermal energy to work (a formulation of the Second Law of Thermodynamics) versus the ’dream’ of a perpetual machine of second kind. It can call attention to misleading interpretations of the Second Law, to its many formulations, some not invoking the concept of entropy, and to the research on dissipative structures that give an understanding of biological organisms and their evolution. The program can also be modified to introduce other resources. As such, it could serve as a complement to teaching programing, optimization techniques and artificial intelligence methods.

Last but not least, our programs were developed using the excellent modeling tools and guides of the Easy Java Simulations and Open Source Physics. These tools provide an easy implementation of the code and graphical objects as well as appealing interactive displays well suited to teaching at undergraduate and graduate levels.

## Data Availability

The original contributions presented in this study are included in the article/Supplementary Materials. Further inquiries can be directed to the corresponding author.

## Supplementary Materials

The following supporting information can be downloaded at https://www.mdpi.com/article/10.3390/e27060565/s1.
